# Antioxidant Effect of *Lonicera caerulea* L. in the Cardiovascular System of Obese Zucker Rats

**DOI:** 10.3390/antiox10081199

**Published:** 2021-07-27

**Authors:** Ezgi Dayar, Martina Cebova, Jan Lietava, Elena Panghyova, Olga Pechanova

**Affiliations:** 1 Centre of Experimental Medicine, Institute of Normal and Pathological Physiology, Slovak Academy of Sciences, 841 04 Bratislava, Slovakia; ezgi.dayar@savba.sk (E.D.); martina.cebova@savba.sk (M.C.); jan.lietava@yahoo.com (J.L.); 2 1st Department of Internal Medicine, Medical Faculty of Comenius University, 811 07 Bratislava, Slovakia; 3 Research Institute of Nutrition, 821 08 Bratislava, Slovakia; Elena.panghyova@nppc.sk

**Keywords:** *Lonicera caerulea* L, heart, aorta, nitric oxide, reactive oxidant species, cholesterol, plasma LDL, blood pressure

## Abstract

*Lonicera caerulea* L. (Loni) represents a promising source of beneficial polyphenols with therapeutical potential in cardiovascular diseases. We aimed to study the effects of Loni and coenzyme Q10 (CoQ10) on selected cardiometabolic parameters and NO/ROS balance in obese Zucker rats. Male Zucker rats were divided into the control group and groups treated with CoQ10 (30 mg/kg/day) or Loni (5 g/kg/day) for 6 weeks. Blood pressure, body weight, heart weight, and plasma lipid profile were determined. NOS activity and protein expressions of eNOS, SOD, NADPH oxidase, and NF-kappa B were measured in the heart and aorta. Neither body weight nor blood pressure were significantly changed after six weeks of Loni or CoQ10 treatment. Both Loni and CoQ10 decreased the plasma LDL level. Moreover, Loni decreased the total cholesterol level. The total NOS activity did not change in the heart after the treatments. However, in the aorta, Loni treatment increased NOS activity and protein expression of SOD and decreased expressions of NADPH oxidase and NF-kappa B compared to both the control and CoQ10 groups. There were no changes in the eNOS protein expression within the groups. In conclusion, it seems that the antioxidant effect of Loni was responsible for both the decrease of plasma LDL and the total cholesterol levels and the increase of vascular NOS activity.

## 1. Introduction

*Lonicera caerulea* L. (Loni) or the blue honeysuckle berry belongs to the Caprifoliaceae family and represents a promising source of beneficial polyphenols with therapeutical potential in cardiovascular and neurodegenerative diseases. Over the centuries it has been used as a traditional medicine in Russia, China, and Japan [1,2]. Loni is rich in vitamin C and polyphenolic compounds such as anthocyanins, phenolic acids, and flavanols. It is unique with the highest vitamin C content and the lowest sugar content among berries. Moreover, it includes minerals like magnesium, phosphorus, calcium, and potassium [2,3,4]. The content of polyphenolics and other compositions of Loni vary depending on harvesting time, cultivation, and climate conditions [5]. The total phenolic content is usually higher at the end of the harvesting time [6]. Blue honeysuckle berries differed significantly in their anthocyanin profile [7]. The most abundant compound is anthocyanin and among them, the predominant anthocyanin is cyanidin-3-glucoside [5,8,9]. Moreover, it has been clearly shown that Loni is an excellent source of iridoids, which are rarely present in other fruits. Containing iridoids, besides anthocyanins, has strengthened the potential biologic activity of Loni, especially the anti-inflammatory effects [8].

Recent studies have indicated that the Loni has antioxidant, anti-inflammatory, neuroprotective, cardioprotective, and antidiabetic effects [5,10]. It acts as a free radical scavenger and decreases the reactive oxygen species (ROS) production. Its anti-inflammatory effects include the inhibition of NF-kappa B activation, reduction of proinflammatory mediators such as TNF-alpha and prostaglandin E2, and overproduction of nitric oxide (NO) [2]. The high content of anthocyanins in Loni has been suggested to be responsible for the modulation of the redox balance by inhibiting ROS and activating antioxidant and detoxifying enzymes [11]. Similarly, iridoids have anti-inflammatory, and hypoglycemic effects by decreasing the upregulation of iNOS and CoX-2 and inhibiting NF-kappa B [12,13].

Since antiobesity and hepatoprotective effects of Loni have been reported [14,15], we aimed to investigate the effects of this berry on the lipid profile, lipid peroxidation, body and heart weight, blood pressure, and NO/ROS balance in the heart and aorta of obese Zucker rats. The effects of Loni were compared with an effective free-radical-scavenging antioxidant coenzyme Q10 (CoQ10).

## 2. Materials and Methods

### 2.1. Chemicals

Most of the chemicals and reagents were obtained from Sigma-Aldrich (Saint Louis, MO, USA); if not, the company is indicated.

### 2.2. Preparation and Characterisation of Lonicera caerulea *L.*

For food preparation, the fresh stoned fruit of Loni originating in the White Carpathians, Slovakia and harvested in June 2019 was mixed with the standard feed and water to modulate the cuboid forms of approximately 3 cm × 3 cm × 3 cm. The created blocks were dried for 6 h at 50 °C to 90% dry weight on a tray dryer.

For the measurement of the total polyphenols and anthocyanins, the stoned fruit was homogenized and 30 g of fruit was extracted in 60 mL of acidified 70% ethanol for 30 min, the extraction was repeated until the extractant had decolorized. Total polyphenols were determined by the Folin–Ciocalteu colorimetric method at 765 nm [16] and the total polyphenol content was calculated as an equivalent of gallic acid with a linearity of 100–800 mg/L, corresponding to an absorbance of 0.1–0.9 (R2 = 0.9954). For the determination of anthocyanins, the AOAC differential pH method [17] in two buffered solutions (KCL buffer pH 1.0 and sodium acetate buffer pH 4.5) was used.

The refractometric method was used for the total sugar determination [18].

### 2.3. Animals and Treatment

Experimental protocols and procedures were approved by the Ethical committee of the Centre of Experimental Medicine, Institute of Normal and Pathological Physiology, Slovak Academy of Sciences according to the European Convention for the Protection of Vertebrate Animals used for Experimental and other Scientific Purpose, Directive 2010/63/EU of the European Parliament.

Twelve-week-old male obese Zucker (fa-/fa-) rats obtained from Charles River, USA were housed in groups of 2 animals, under a 12 h light–12 h dark cycle, at a constant humidity (45–65%) and temperature (20–22 °C).

Obese Zucker (fa-/fa-) rats were divided into the control group (*n* = 6) and groups treated with CoQ10 (*n* = 6) or Loni (*n* = 6) for six weeks. Control and CoQ10 groups were fed with a standard diet ad libitum. The Loni group was fed with a special diet that contained dry fruit of Loni (5 g/kg/day) mixed with a standard diet (30 g/day). CoQ10 in the dose of 30 mg/kg/day was administered via the drinking water.

Blood pressure was measured by tail-cuff plethysmography every week. At the end of the treatment, the animals were sacrificed; heart weight (HW) and tibia length (TL) were determined. Relative heart weight was calculated as a HW/TL ratio. Blood plasma were collected for measuring the levels of triglyceride, total cholesterol, HDL, and LDL. Samples of the heart and aorta were used to determine NOS activity and eNOS, SOD, NF-kappaB, and NADPH oxidase protein expressions. The conjugated diene level was analyzed in the heart.

### 2.4. Plasma Lipid Levels

The triglyceride, total cholesterol, HDL, and LDL levels were measured in the plasma by commercially available kits.

### 2.5. Total NOS Activity

Total NOS activity was determined in crude homogenates of the heart and aorta by measuring the formation of [3H]-L-citrulline from [3H]-L-arginine (ARC, Saint Louis, MO, USA). [3H]-L-citrulline was measured with the Quanta Smart TriCarb Liquid Scintillation Analyzer (Packard Instrument Company, Meriden, CT, USA) [19].

### 2.6. Western Blot Analysis

Tissue samples of the heart and aorta were homogenized in 0.5 mM Tris lysis buffer containing the protease inhibitor cocktail (Sigma-Aldrich, Saint Louis, MO, USA), centrifugated (15,000 rpm at 4 °C for 20 min), and protein concentrations were determined by the Lowry protein assay. Western blot was performed as a following protocol: electrophoresis, transfer to the membrane, blocking, overnight primary antibody incubation, incubation with the secondary antibody, and visualization. Supernatants were subjected to SDS-PAGE using 12% gels, proteins were transferred to nitrocellulose membranes and blocked with 5% non-fat milk in the Tris-buffer solution (TBS; pH 7.6) containing 0.1% Tween-20 (TBS-T) for 1 h at room temperature and incubated with a primary polyclonal rabbit anti-eNOS (1:1000, Abcam, ab5589), anti-SOD 1 (1:2000, Abcam, ab16831), anti-NADPH oxidase 4 (1:2000, Abcam, ab154244), anti-NF-kappa B p65 (1:1000, Abcam, ab16502) antibodies, anti-GAPDH (1:5000, Abcam, ab201822), and anti-β-actin (1:2000, Abcam, ab8227) as a loading control overnight. Antibodies were detected using a secondary peroxidase-conjugated goat anti-rabbit antibody (1:5000, Abcam, ab97051) by vortexing at the room temperature for 2 h. The intensity of bands was visualized using the enhanced chemiluminescence system (ECL, Amersham, UK), quantified by using the ChemiDocTM Touch Imagine System (Image LabTM Touch software, BioRad, Hercules, CA, USA) and normalized to GAPDH bands for the heart and β-actin bands for the aorta.

### 2.7. Concentration of the Conjugated Dienes

The lipid extracts of the hearts were used for the determination of the conjugated diene concentrations. The samples were homogenized in 15 mmol/dm^3^ EDTA containing 4% NaCl. Lipids were extracted using a 1:1 chloroform–methanol mixture. Chloroform was evaporated in the N2 atmosphere and after the addition of cyclohexane, conjugated diene concentrations were determined spectrophotometrically (λ = 233 nm, NanoDrop 2000c, UV–Vis spectrophotometer).

### 2.8. Data Analysis

A one-way analysis of variance (ANOVA) and Bonferroni test were used for statistical analysis. Values were considered significant with probability value *p* < 0.05 (both for the ANOVA and Bonferroni test). *p* values were multiplicity adjusted. F values are given under the tables and figures. Data are presented as mean ± SEM.

## 3. Results

### 3.1. Characterization of Lonicera caerulea *L.*

The content of the total polyphenols, anthocyanins, and total sugars of Loni is shown in the Table 1.

### 3.2. Body Weight, Relative Heart Weight, and Blood Pressure

Neither body weight nor blood pressure were significantly changed after six weeks of treatments within all groups. On the other hand, relative heart weight was decreased in the Loni group (Table 2).

### 3.3. Plasma Lipid Profile

Both Loni and CoQ10 decreased the plasma LDL level. Moreover, Loni decreased the total cholesterol level (Table 3).

### 3.4. Total NOS Activity

**Figure 1:**
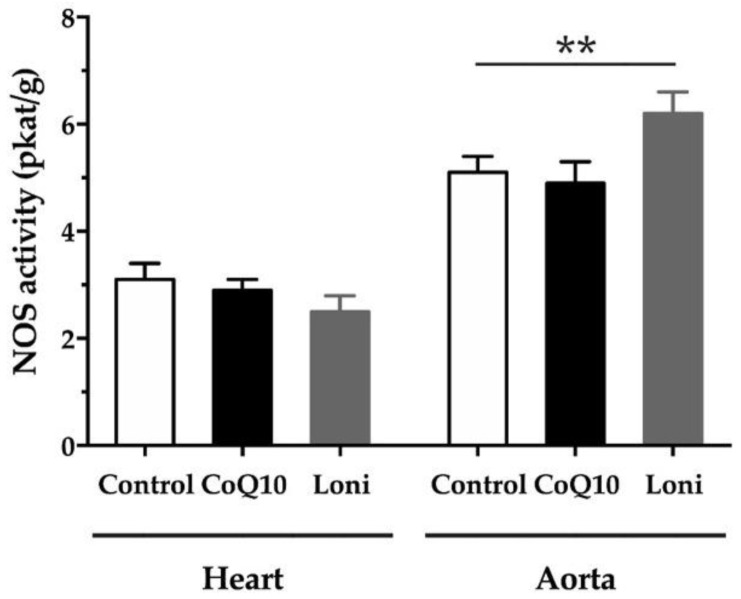
Nitric oxide synthase (NOS) activity in the heart and aorta of the obese Zucker rats treated with coenzyme Q10 (30 mg/kg/day) and *Lonicera caerulea* L. (5 g/kg/day). CoQ10; coenzyme Q10, Loni; *Lonicera caerulea* L. Heart; F (2.15) = 0.92483, *p* = 0.41807; aorta; F (2.15) = 6.0098, *p* = 0.01211. ** *p* < 0.01 compared to the control group. Data are means ± SEM from 6 animals in each group.

Neither the CoQ10 nor Loni treatment changed the NOS activity in the heart significantly. However, in the aorta, Loni was able to increase NOS activity. There was no significant change in the CoQ10 group (Figure 1).

### 3.5. Protein Expressions of eNOS, SOD, NADPH Oxidase, and NF-kB

**Figure 2:**
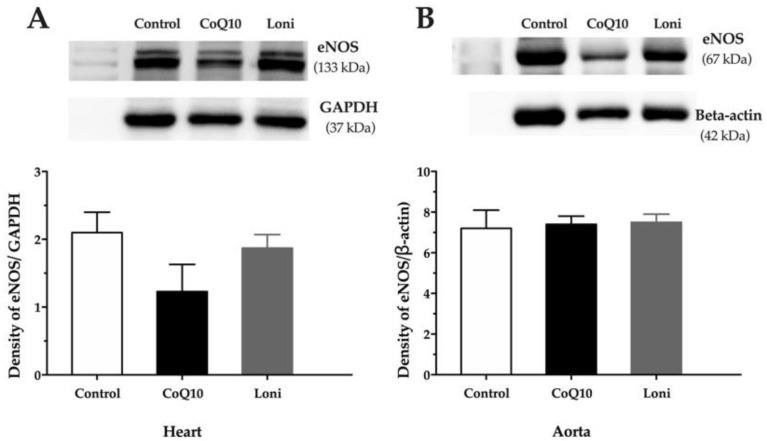
eNOS protein expression in the heart (**A**) and aorta (**B**). CoQ10; coenzyme Q10, Loni; *Lonicera caerulea* L. Heart; F (2.15) = 1.4312, *p* = 0.26986; aorta; F (2.15) = 0.04720, *p* = 0.95404. Data are means ± SEM from 6 animals in each group.

**Figure 3:**
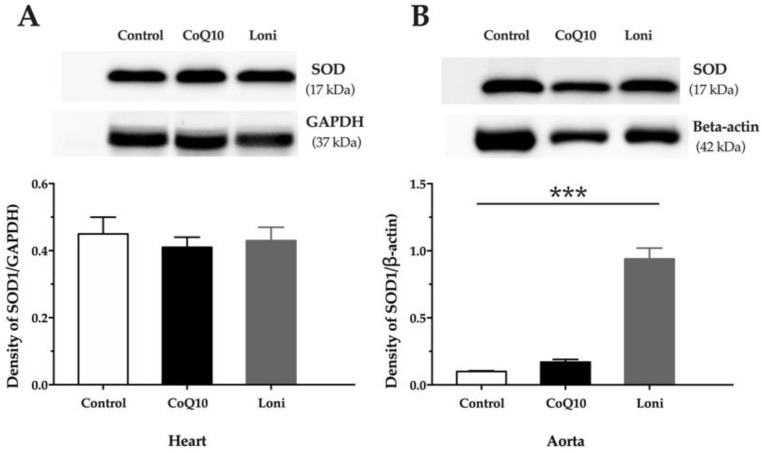
SOD1 protein expression in the heart (**A**) and aorta (**B**). CoQ10; coenzyme Q10, Loni; *Lonicera caerulea* L. Heart; F (2.15) = 0.25449, *p* = 0.77859; aorta; F (2.15) = 85.812, *p* = 0.00000. *** *p* < 0.001 compared to the control group. Data are means ± SEM from 6 animals in each group.

**Figure 4:**
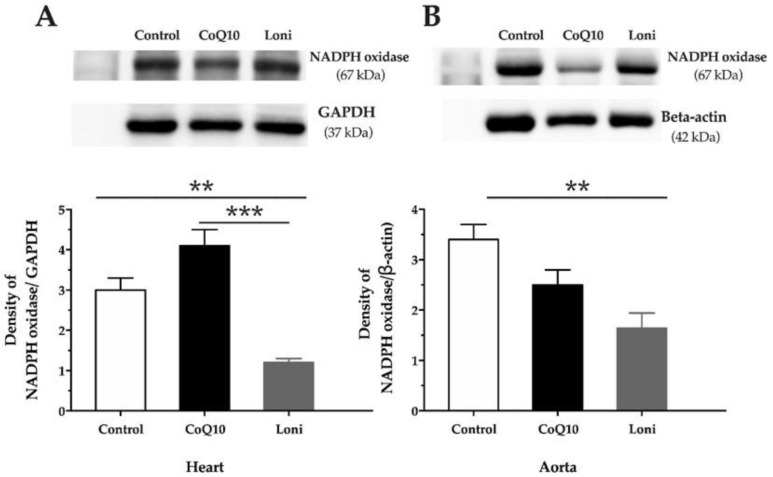
NADPH oxidase protein expression in the heart (**A**) and aorta (**B**). CoQ10; coenzyme Q10, Loni; *Lonicera caerulea* L. Heart; F (2.15) = 23.100, *p* = 0.00003; aorta; F (2.15) = 9.4086, *p* = 0.00225. ** *p* < 0.01 compared to the control group, *** *p* < 0.001 compared to the coQ10 group. Data are means ± SEM from 6 animals in each group.

**Figure 5:**
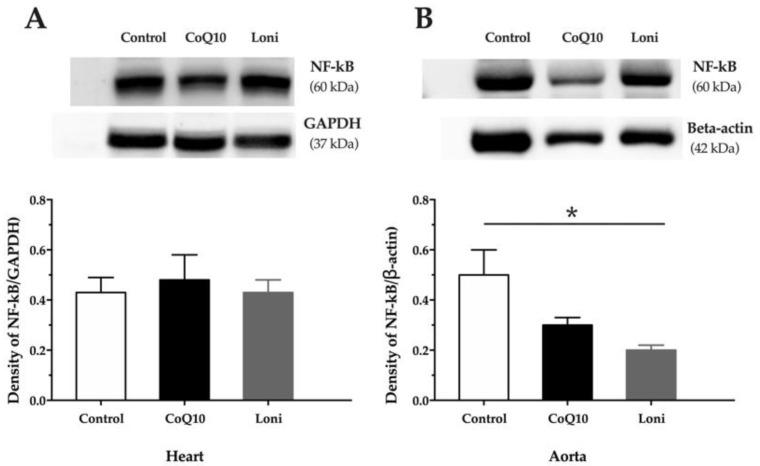
NF-kB protein expression in the heart (**A**) and aorta (**B**). CoQ10; coenzyme Q10, Loni; *Lonicera caerulea* L. Heart; F (2.15) = 0.15881, *p* = 0.85457; aorta; F (2.15) = 12.409, *p* =0.00066. * *p* < 0.05 compared to the control group. Data are means ± SEM from 6 animals in each group.

There were no significant changes in the eNOS protein expressions in both the heart and aorta after CoQ10 or Loni treatment (Figure 2A,B). Loni markedly increased SOD protein expression in the aorta, while there were no significant changes in the heart (Figure 3A,B). CoQ10 did not change SOD protein expression in the tissues (Figure 3A,B). Loni treatment was able to decrease NADPH oxidase protein expressions in the heart and aorta. On the other hand, there were no significant changes after CoQ10 treatment (Figure 4A,B). Both CoQ10 and Loni decreased NF-kappa B protein expressions in the aorta, while there were no significant changes in the heart (Figure 5A,B).

### 3.6. Conjugated Diene Concentrations

**Figure 6:**
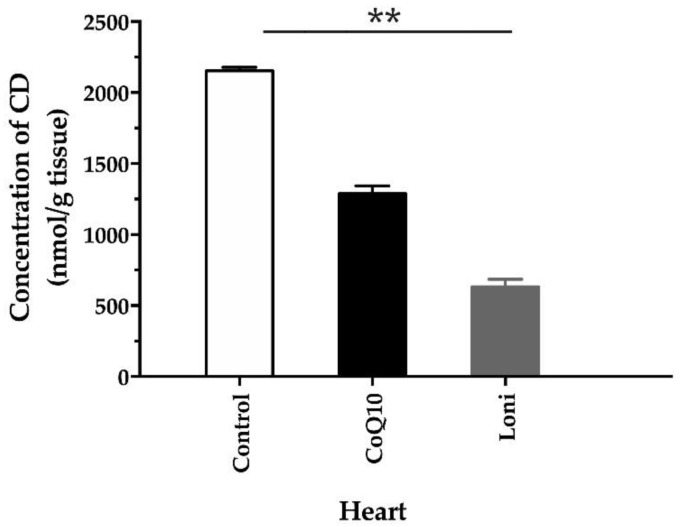
Conjugated diene concentrations in the heart. CoQ10; coenzyme Q10, Loni; *Lonicera caerulea* L. CD; F (2.15) = 275.93, *p* = 0.00000. ** *p* < 0.01 compared to the control group. Data are means ± SEM from 6 animals in each group.

In the heart, CD concentration decreased significantly only after the Loni treatment (Figure 6).

## 4. Discussion

The effect of *Lonicera caerulea* L. on selected cardiometabolic parameters and the production of reactive oxygen species and nitric oxide in obese Zucker (fa-/fa-) rats were studied and compared with a commercially used antioxidant—coenzyme Q10. It is well known that the deficiency of CoQ10 is related to different cardiometabolic diseases such as dyslipidemia, diabetes mellitus, and atherosclerosis, but also to muscular dystrophy, and others. Administration of CoQ10 helps in the protection against oxidative stress in cardiovascular diseases, type 2 diabetes, and metabolic syndrome [20]. It has been shown that CoQ10 may improve antioxidant capacity and decrease oxidized-LDL induced generation of ROS, downregulation of eNOS, and upregulation of iNOS [21]. In the obese Zucker rats used in our study, CoQ10 decreased the plasma LDL level but had no effect on the total cholesterol. Loni was able to decrease both the plasma LDL and total cholesterol levels significantly.

Similarly, Loni decreased the total plasma cholesterol and increased the content of HDL in both high-fructose and high-fed-induced hyperlipidemic rats. In the same models it normalized levels of plasma triglycerides and glucose [9,22,23]. Additionally, 12-week oral administration of Loni displayed a dose-dependent decrease in the serum insulin levels, HbA1c contents, and blood glucose levels in high-fat-induced mild diabetic mice [24]. Loni inhibited high fed diet-induced hepatic lipid peroxidation by improving the insulin sensitivity and Nrf2-mediated antioxidant pathway in mice [15]. In a double-blind, counterbalanced, crossover intervention study, the consumption of Loni significantly lowered the diastolic blood pressure and heart rate in older adults [25]. In our study, the tendency of reduction in blood pressure after the Loni treatment was not however significant.

Nevertheless, the tendency in blood pressure reduction could be caused by increased vascular NO synthase activity after the Loni treatment. Several studies have documented that berry anthocyanins are able to induce NO production by upregulating the expression of eNOS. Cyanidin-3-glucoside, the most abundant anthocyanin in the berries, can improve vascular endothelial function by triggering eNOS phosphorylation [26,27]. However, in our study using obese Zucker rats, we did not observe increased protein expression of eNOS in either the aorta or the heart after the Loni treatment. On the other hand, Loni treatment increased the protein expression of SOD and decreased expressions of NADPH oxidase and NF-kappa B compared to both the control and CoQ10 groups. Similarly, Jin et al. [28] have reported that the treatment with Loni suppressed lipopolysaccharide (LPS)-induced activation of NF-kappaB and elevation of TNF-alpha. Moreover, it inhibited expression of iNOS and COX-2 and their products, NO and PGE2, in LPS-stimulated RAW264.7 cells [28]. In LPS-stimulated human macrophages, Rupasinghe et al. [29] also reported that Loni extracts significantly inhibited expression of the major proinflammatory cytokines such as interleukin-6, TNF-alpha, PGE2, and a COX-2 enzyme [29]. In Loni extract-fed adjuvant-induced arthritis Sprague-Dawley rats, serum levels of proinflammatory biomarkers including interleukin-6, TNF-alpha, and NO were significantly reduced [30]. Cyanidin-3-O-glucoside has been shown to suppress LPS-stimulated TNF-alpha and interleukin-6 mRNA and protein expression and block phosphorylation of NF-kappaB in LPS-stimulated macrophages [31]. In fibroblast cells, treatment with Loni inhibited LPS-induced inflammatory factors such as interleukin-1b, interleukin-6, and TNF-alpha and oxidative damage by reducing ROS production and lipid peroxidation [32]. Similarly, our results documented a decreased concentration of a marker of lipid peroxidation—the conjugated dienes in the heart after the Loni treatment.

The antioxidant effects of Loni and anthocyanin treatments have been demonstrated also in rat cortical cells by protecting against glutamate-induced toxicity [33] or in the animal models of Alzheimer disease by the regulation of the phosphorylated-phosphatidylinositol 3-kinase-Akt-glycogen synthase kinase 3 beta pathway [34].

Taken together, in obese Zucker rats, Loni treatment did not increase eNOS protein expression, thus it seems that the antioxidant effect of Loni was responsible for both a decrease of plasma LDL and total cholesterol levels and an increase of vascular NOS activity. Since anthocyanins were a major component of polyphenols in our study as well, we hypothesized that these substances were mainly responsible for the antioxidant effects of *Lonicera caerulea* L.

## 5. Conclusions

The antioxidant effect of *Lonicera caerulea* L. was demonstrated in several animal models of cardiometabolic diseases. We first demonstrated this effect in obese Zucker rats. Since *Lonicera caerulea* L. is rich in antioxidant anthocyanins and low in sugar, which was demonstrated also in our study, this berry can be suggested as a supplement treatment in dyslipidemia and other cardiometabolic disorders.

## Figures and Tables

**Table 1:** Total polyphenols, anthocyanins, and sugars in the stoned fresh fruit of *Lonicera caerulea* L. (Loni).

	Total Polyphenols (mg/kg)	Total Anthocyanins (mg/kg)	Total Sugars (g/kg)
Loni	576.0 ± 61.2	135.0 ± 18.5	73.2 ± 9.5

**Table 2:** The overview of body weight (BW), heart weight (HW)/tibia length (TL) ratio, and blood pressure (BP) in the control, coenzyme Q10 (CoQ10), and *Lonicera caerulea* L. (Loni) groups.

	BW (g)	HW/TL (×10^−2^)	BP (mmHg)
Control	698.5 ± 20.4	3.2 ± 0.1	147 ± 2.5
CoQ10	639.8 ± 42.3	3.1 ± 0.1	142 ± 2.3
Loni	669 ± 40.9	2.8 ± 0.06 **	136 ± 2.9

BW; F (2.15) = 3.49, *p* = 0.057; HW/TL; F (2.15) = 6.64, *p* = 0.009; BP; F (2.15) = 3.30, *p* = 0.065. ** *p* < 0.01 compared to the control group. Data are means ± SEM from 6 animals in each group.

**Table 3:** Lipid profile of the control, coenzyme Q10 (CoQ10), and *Lonicera caerulea* L. (Loni) groups.

	TG (mmol/L)	CHOL (mmol/L)	HDL (mmol/L)	LDL (mmol/L)
Control	2.87 ± 0.21	7.65 ± 0.18	147.3 ± 10.1	70.9 ± 2.7
CoQ10	2.91 ± 0.48	6.23 ± 0.52	143.2 ± 6.3	49.6 ± 4.1 *
Loni	1.96 ± 0.2	4.9 ± 0.4 *	153.03 ± 6.9	40.2 ± 2.1 ***

TG; triglyceride, CHOL; total cholesterol, HDL; high-density lipoprotein, LDL; low-density lipoprotein. TG; F (2.15) = 2.7375, *p* = 0.09694; CHOL; F (2.15) = 11.620, *p* = 0.00090 *; HDL; F (2.15) = 0.38813, *p* = 0.68494; LDL; F (2.15) = 25.937, *p* = 0.00001. * *p* < 0.05; *** *p* < 0.001 compared to the control group. Data are means ± SEM from 6 animals in each group.

## Data Availability

Data is contained within the article.
